# Silencing cytokeratin 18 gene inhibits intracellular replication of *Trypanosoma cruzi *in HeLa cells but not binding and invasion of trypanosomes

**DOI:** 10.1186/1471-2121-9-68

**Published:** 2008-12-17

**Authors:** Carla Claser, Marli Curcio, Samanta M de Mello, Eduardo V Silveira, Hugo P Monteiro, Mauricio M Rodrigues

**Affiliations:** 1 Centro Interdisciplinar de Terapia Gênica (CINTERGEN), Universidade Federal de São Paulo-Escola Paulista de Medicina, Rua Mirassol, 207, São Paulo-SP, 04044-010, Brazil; 2 Departamento de Microbiologia, Imunologia e Parasitologia, Universidade Federal de São Paulo-Escola Paulista de Medicina, Rua Botucatuo, 862, Sao Paulo-SP, 04023-062, Brazil; 3 Singapore Immunology Network, Agency for Science, Technology and Research (A*Star), Biopolis, Singapore; 4 Departamento de Bioquímica e Biologia Molecular, Universidade Federal de São Paulo-Escola Paulista de Medicina, Rua 3 de Maio, 100, São Paulo-SP, 04044-020, Brazil; 5 Singapore Immunology Network, Agency for Science, Technology and Research (A*Star), Biopolis, Singapore

## Abstract

**Background:**

As an obligatory intracellular parasite, *Trypanosoma cruzi*, the etiological agent of Chagas' disease, must invade and multiply within mammalian cells. Cytokeratin 18 (CK18) is among the host molecules that have been suggested as a mediator of important events during *T. cruzi*-host cell interaction. Based on that possibility, we addressed whether RNA interference (RNAi)-mediated down regulation of the CK18 gene could interfere with the parasite life cycle *in vitro*. HeLa cells transiently transfected with CK18-RNAi had negligible levels of CK18 transcripts, and significantly reduced levels of CK18 protein expression as determined by immunoblotting or immunofluorescence.

**Results:**

CK18 negative or positive HeLa cells were invaded equally as well by trypomastigotes of different *T. cruzi *strains. Also, in CK18 negative or positive cells, parasites recruited host cells lysosomes and escaped from the parasitophorous vacuole equally as well. After that, the growth of amastigotes of the Y or CL-Brener strains, was drastically arrested in CK18 RNAi-treated cells. After 48 hours, the number of amastigotes was several times lower in CK18 RNAi-treated cells when compared to control cells. Simultaneous staining of parasites and CK18 showed that in HeLa cells infected with the Y strain both co-localize. Although the amastigote surface protein-2 contains the domain VTVXNVFLYNR previously described to bind to CK18, in several attempts, we failed to detect binding of a recombinant protein to CK-18.

**Conclusion:**

The study demonstrates that silencing CK18 by transient RNAi, inhibits intracellular multiplication of the Y and CL strain of *T. cruzi *in HeLa cells, but not trypanosome binding and invasion.

## Background

The protozoan parasite *Trypanosoma cruzi *is the etiologic agent of Chagas' disease, which chronically affects more than 10 million individuals in the Americas, with an annual death toll of approximately 45,000 people [1]. In spite of the significant reduction in transmission over the past twenty years in countries such as Argentina, Brazil, Chile and Uruguay, Chagas' disease is still a major health problem for many Latin American countries. A recent epidemiological study in dogs also revealed a dangerously widespread epidemic in the state of Texas [2].

The efficacy of conventional chemotherapy with nifurtimox or benznidazole is low and varies greatly according to the infection status of the patients (acute or chronic) and the parasite strains. Although new drugs could potentially solve the problem, Chagas' disease is one of the most neglected diseases in terms of drug development with a single new compound being tested in phase 1/2 trials (reviewed in ref. [3]). Based on these poor short term prospects, studies aimed at understanding the requirements for parasite invasion and multiplication of *T. cruzi *are still relevant.

Invasion and multiplication within a variety of host cells are critical features for the survival of *T. cruzi*. The invasion process of mammalian non-phagocytic cells has attracted a great deal of interest in the past 20 years and notable advances have been obtained. Different routes of invasion described by researchers have been mediated by distinct cell surface receptors, secondary messengers and transcription factors (recently reviewed in refs. [4-6]). In one of the mechanisms described, the infective forms of *T. cruzi *(trypomastigotes) take advantage of a host cell mechanism for puncture repair and exocytosis of lysosomes to recruit these organelles to the parasite-cell junction. After a Ca^2+ ^dependent fusion with the cell membrane, lysosomes become part of the intracellular parasitophorous vacuole. Alternatively, parasites can use a lysosome-independent pathway. In this case, the host cell plasma membrane accounts for the parasitophorus vacuole. Ultimately, these organelles fuse with lysosomes, a step that recently has been shown to be critical for parasite retention and productive infection [7]. Among the parasite surface molecules that might be relevant for the attachment, signaling and penetration are members of a family of mucin-like glycoproteins, members of a *trans*-sialidases family, and proteases (reviewed in ref. [8-10]).

Once inside the host cells, *T. cruzi *manages to disrupt the parasitophorus vacuole, an event that could be mediated by lytic proteins and the enzyme *trans*-sialidase [11-14]. During the escape, trypomastigotes transform into amastigotes and express on its surface some stage-specific molecules [15-17]. In the host cell cytoplasm, GPI-deficient amastigotes arrest replication and subsequently failed to differentiate into trypomastigotes [18]. The molecular events required for amastigote transformation into trypomastigotes and their release from the infected cells are completely unknown.

The accessibility of the new reverse genetic tools such as RNAi have allowed investigators to search for host molecules that might be important for the *T. cruzi *invasion as well as intracellular parasite survival and multiplication. Villalta's group demonstrated that the silencing of laminin-γ1 expression, a protein belonging to the extracellular matrix and regulated by the parasite, by cultured human coronary artery smooth muscle cells rendered them significantly more resistant to the binding and penetration of parasites (reviewed in [5,6,19]). This observation supports the hypothesis that the binding to laminin is an important step for the early process of *T. cruzi *infection. Also, it suggests that laminin-binding proteins expressed on the surface of *T. cruzi *are important virulence factors during the infection of human smooth muscle cells [5,6,19,20]. Utilizing a similar approach, the same group demonstrated that stable interference of thrombospondin-1 expression, also a protein belonging to the extracellular matrix, in cultured HeLa cells *in vitro *caused an increase in the cell resistance to *T. cruzi *invasion [5,6,21]. These studies demonstrated the effectiveness of the RNAi strategy when studying the host cell factors critical for parasite survival and eventually identifying potential targets for new therapies.

One of the laminin-binding proteins, a member of the family of the *trans*-sialidases of *T. cruzi *(Tc-85-11), was shown to bind to host cytokeratin 18 (CK18). The CK18 is an intermediate filament protein of the acid cytokeratin family belonging to type I, expressed in the internal epithelia cell cytoplasm. This protein together with CK8 exhibit resistance features in response to other forms of stress and to apoptosis [22]. This interaction through a C-terminal peptide ligand denominated FLY domain (VTVXNVFLYNR, [23]) was shown to be relevant for parasite invasion as anti-cytokeratin antibodies inhibited the infection of epithelial cells by *T. cruzi*. The FLY motif is not only present in proteins expressed on the surface of trypomastigotes but also on a protein that is abundant on the surface of amastigotes denominated Amastigote Surface Protein-2 (ASP-2, [24,25]). Based on this observation, it was our intention to further investigate whether the host cell CK18 expression was indeed critical for *T. cruzi *invasion and/or subsequent parasite growth *in vitro*. For this purpose we used HeLa cells in which we transiently down-regulated the host CK18 using the RNAi strategy. Upon infection, we evaluated parasite attachment, penetration, lysosome recruitment, phagolysosomal escape and cytoplasm replication in cultured HeLa cells. RNAi-mediated knock-down of CK18 did not interfere with trypomastigotes mediated events. Once amastigotes from two distinct strains of *T. cruzi *were in the cytoplasm of CK18 RNAi-treated cells, their development was severely inhibited.

## Results

### RNAi knockdown of endogenous CK18 in cultured HeLa cells

**Figure 1:**
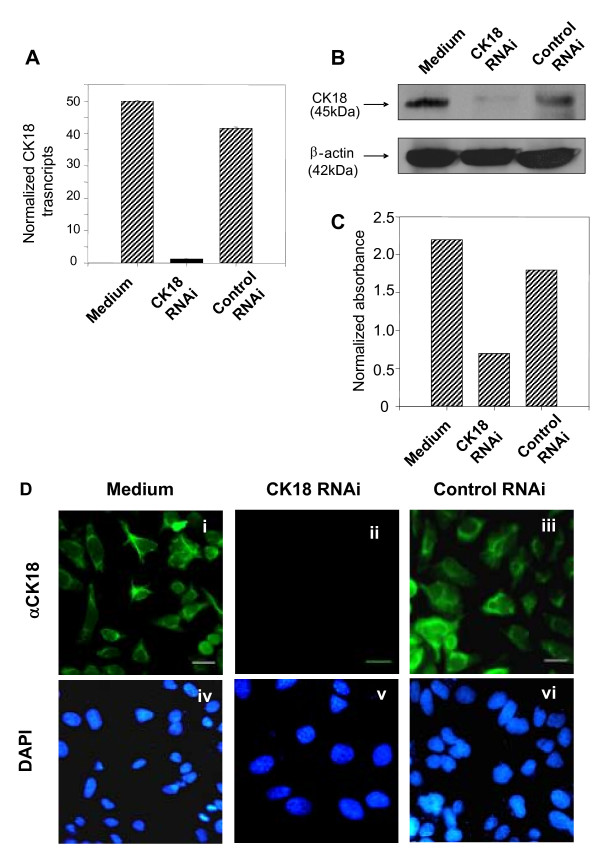
**CK18 expression on RNAi-treated HeLa cells**. (A) HeLa cells were left untreated (Medium) or transfected with the CK18 or control RNAi as described in the Methods section. CK18 transcripts were estimated by Real-time PCR. Results are expressed in arbitrary units after normalization with RPL13 transcritps. Results are represented as an average of triplicate samples ± SD. The experiments were performed three times with similar results. (B) Immunoblot analysis of HeLa cells extracts untreated (Medium) or treated with CK18 or control RNAi. Stainings were performed with MAbs specific for CK18 or β-actin. (C) Densitometry of immunoblot was performed and the bars represent the absorbance of CK18 bands after normalization using the staining with anti-β-actin. (D) IFA staining of HeLa cells untreated (Medium) or treated with CK18 or control RNAi. HeLa cells were treated with RNAi for 24 h and infected with tissue culture trypomastigotes for 48 h. After that period, cells were stained with a MAbs specific for CK18 (green) and DAPI (blue). Magnification bar = 35 μM.

Earlier studies have suggested that CK18 plays a role during the interaction between trypomastigotes of *T. cruzi *and non-phagocytic mammalian cells [23,26]. To further evaluate whether CK18 expression was indeed critical for the host parasite interaction, we established an *in vitro *transient transfection system to knock down the expression of endogenous CK18. Following the transfection of cultured HeLa cells with CK18 RNAi, we evaluated the levels of CK 18-specific transcripts with the aid of quantitative real-time PCR. As shown in Fig. 1A, 24 h following transfection, the amount of CK18 transcripts were reduced by more than 40 fold in HeLa cells treated with CK18 RNAi when compared to control cells untreated or treated with a control RNAi. The treatment did not modify the expression of the control housekeeping gene RLP13 used to normalize the level transcript on each sample. We then studied whether the reduction of CK18 transcripts by RNAi were reflected at the protein level by quantitative immunoblotting analysis (Fig. 1B). As estimated by densitometry, the inhibition of CK18 expression was ~70%, without significant inhibition on the synthesis of β-actin (Fig. 1C). Finally, we performed an immunofluorescence based assay to confirm that CK18 expression was inhibited in most cells. As shown in Fig. 1D, HeLa cells transiently transfected with CK18 RNAi and subsequently infected with trypomastigotes of *T. cruzi*, had no detectable fluorescence (Fig. 1Dii). Significant inhibition of CK18 expression in cultured HeLa cells was maintained for at least 72 h following transfection (data not shown).

**Figure 2:**
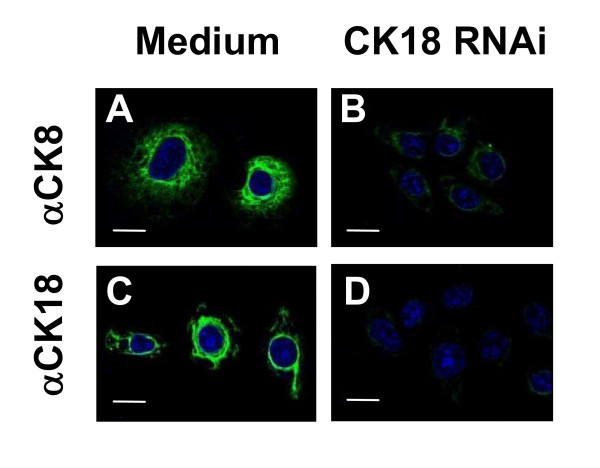
**Inhibition of CK8 expression in CK18 RNAi-treated HeLa cells**. HeLa cells were treated with medium or transfected with the CK18 RNAi as described in the Methods section. After 24 h, cells were stained with a MAbs specific for CK8 or CK18 (green) and DAPI (blue). Magnification bar = 20 μM.

Because CK18 is part of intermediate cell filaments which also contains large amounts of CK8, we tested whether CK18 RNAi-treated HeLa cells would also reduce the expression of stable CK8. Indeed, CK18 RNAi-treatment reduced the immunofluorescence of HeLa cell stained with specific anti-CK8 antibodies (Fig 2B). Similar experiments were performed with anti-CK19. In this case we could not detect CK19 in untreated or CK18 RNAi-treated HeLa cells (data not shown).

**Figure 3:**
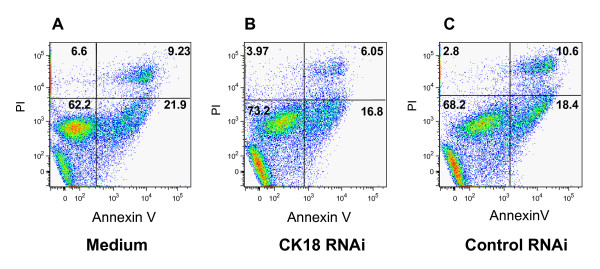
**CK18 RNAi treatment does not cause apoptosis of HeLa cells**. HeLa cells were left untreated (Medium) or transfected with CK18 or control RNAi. After 34 h, cells were labeled with propidium iodine (PI) and Annexin-V-FITC. Cells were analyzed by FACS.

Also, it was possible that the reduction of the levels of this protein could interfere with cell survival. To evaluate this possibility, HeLa cells, transfected or not, were kept in culture for 34 h before cell viability was estimated by staining with Annexin-V-FITC and PI. As shown in Fig. 3, we did not observe an increase in the frequency of Annexin-positive cells when we compared CK18 RNAi-treated or untreated cells. To confirm Annexin assay results, we measured the mitochondrial activity by MTT assay, a method for assessing cell viability by quantifying the conversion of the tetrazolium salt to its formazan product. We observed that there was no difference between the CK18 RNAi- treated or control cells (containing only medium or control RNAi, data not shown). This demonstrates that RNAi-mediated CK18 knockdown did not promote apoptosis.

### RNAi-mediated CK18 knockdown impairs amastigote replication in the cytoplasm of HeLa cells

**Figure 4:**
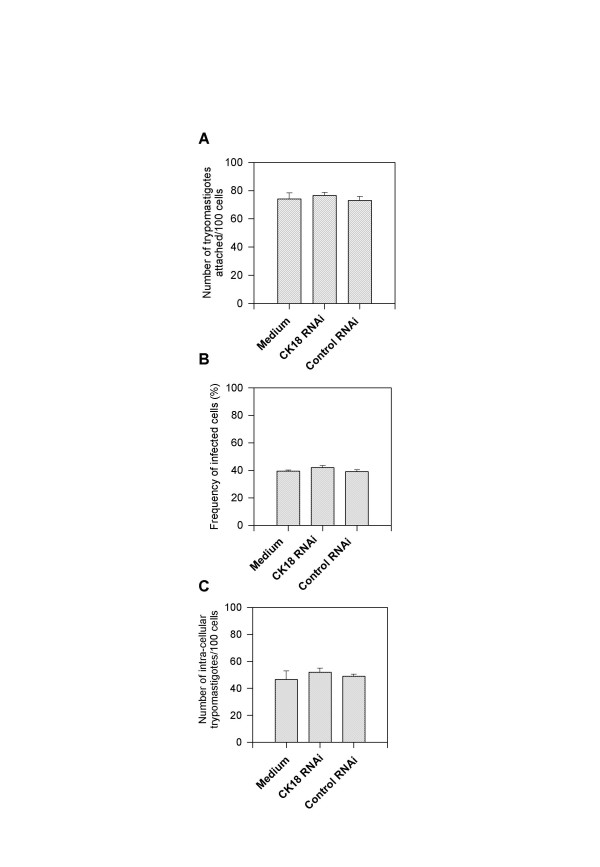
**CK18 RNAi treatment does not inhibit trypomastigote attachment or invasion of HeLa cells**. HeLa cells were left untreated or treated with CK18 RNAi or control RNAi. After 24 h, cells were infected with culture trypomastigotes of the Y strain of *T. cruzi*. (A) Number of trypomastigotes attached to HeLa cells; (B) Frequency of infected HeLa cells; (C) Number of parasites per 100 cells. Bars represent the average of triplicate cultures ± SD.

**Figure 5:**
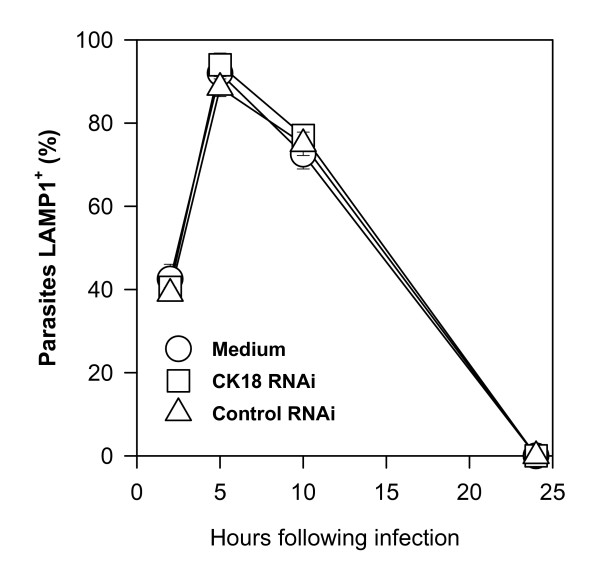
**CK18 RNAi treatment does not inhibit lysosome recruitment to the parasitophorous vacuole and parasite escape to cytoplasm**. HeLa cells were left untreated or treated with CK18 RNAi or control RNAi. After 24 h, cells were infected with culture trypomastigotes of the Y strain of *T. cruzi*. The frequency of parasites stained with anti-LAMP-1 MAb is shown at the indicated hours post-infection. Dots represent the average of triplicate cultures ± SD.

CK18 RNAi-treated HeLa cells were initially infected with tissue culture trypomastigotes of the Y strain of *T. cruzi*. After 2 h, cells were washed and fixed. The number of trypomastigotes attached, the frequencies of infected cells, as well as the number of trypomastigotes inside 100 cells were estimated. We found that all of them were similar in CK18 RNAi-treated or untreated cells (Fig. 4A, B and 4C, respectively). We therefore concluded that the reduction of the CK18 expression did not change the ability of trypomastigotes to attach and invade these cells. During the invasion process, the parasites recruit lysosomes, which fuse with the parasitophorous vacuoles containing trypomastigotes. Approximately 5 h after infection, more than 90% of the parasites can be co-stained with antibodies specific for the phagolysosomal marker LAMP-1 (Fig. 5). After that period, trypomastigotes differentiate and escape the parasitophorous vacuoles/phagolysosomes. We estimated whether both processes also occur in CK18 RNAi-treated cells. At different times post-infection, the frequency of parasites co-stained with the phagolysosomal marker LAMP-1 was similar among infected HeLa cells, regardless of whether they were transfected with CK18 RNAi (Fig. 5).

Twenty-four hours after infection, no parasites could be co-stained with anti-LAMP-1 whether they were in the cytoplasm of CK18 RNAi-treated or non-treated cells. We then concluded that the reduction of the CK18 expression did not influence the ability of parasites to recruit the host cell lysosomes, transform into amastigotes and exit the parasitophorous vacuoles.

**Figure 6:**
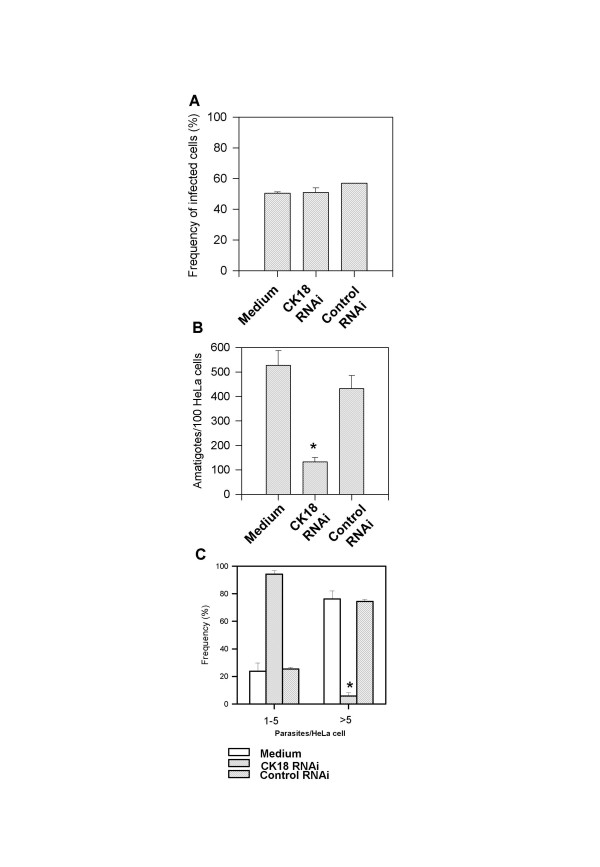
**CK18 RNAi treatment inhibit amastigotes multiplication in HeLa cells**. HeLa cells were left untreated or treated with CK18 RNAi or control RNAi. After 24 h, cells were infected with culture trypomastigotes of the Y strain of *T. cruzi*. Forty-eight hours post infection, we estimated: (A) The frequency of infected HeLa; (B) The number of parasites per 100 cells; (C) The frequency of infected cells with 1–5 or > 5 parasites. Bars represent the average of triplicate cultures ± SD. Asterisks denote significantly lower numbers when compared to non-transfected or control RNAi (P < 0.01).

After 24 h, amastigotes were in the host cell cytoplasm where they began multiplying. When we estimated the frequency of infected cells 48 h after infection, we found that both CK18 RNAi-treated cells and non-treated HeLa cells had similar percentages of infected cells (Fig. 6A). Nevertheless, the number of intracellular amastigotes per 100 HeLa cells treated with CK18 RNAi was 79% lower. Considering that the frequencies of infected cells are lower, we estimated an inhibition of 85% on the amastigote growth during the 24 h period (from 24 to 48 h after infection, Fig. 6B). The impact of CK18 RNAi treatment on parasite growth can be further highlighted by our estimates on the frequency of infected cells containing 1 to 5 or more than 5 parasites. In the case of CK18 RNAi-treated HeLa cells, less than 10% of the infected cells had more than 5 parasites. In contrast, more than 75% of the infected control cells were infected with more than 5 amastigotes per cell (Fig. 6C). Also, we tested the effect of CK18 RNAi treatment on parasite growth in LLC-MK2 cells, after infection with trypomastigotes of Y strain. As observed in HeLa cells, the amastigotes growth during the 24 h period (from 24 to 48 h after infection) was dramatically reduced in the LLC-MK2 cells. While 70% of the CK18 RNAi-treated LLC-MK2 cells displayed with 1 to 5 parasites, 80% of control RNAi-treated infected cells had > 5 amastigotes per cell (data not shown). This suggests that the CK18 RNAi treatment can also impair the amastigotes growth in LLC-MK2 cells.

**Figure 7:**
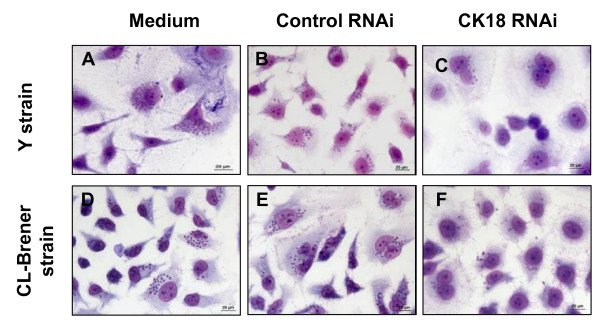
**Analysis of amastigote replication on CK18 RNAi treated HeLa cells infected with different *T. cruzi *strains**. HeLa cells were left untreated (A and D), or were transfected with control RNAi (B and E), or CK18 RNAi (C and F). These cells were then infected with tissue culture trypomastigotes of the Y (A-C) or CL-Brener (D-F) strains of *T. cruzi*. After 48 h, cells were fixed and stained with Giemsa. Magnification bar = 20 μM.

We then evaluated whether CK18 expression in HeLa cells was in fact important for the multiplication of amastigotes of different *T. cruzi *strains. To answer this question, CK18 RNAi-treated HeLa cells were infected with trypomastigotes of Y or CL-Brener strains. After 48 h, the frequency of infected cells and the number of amastigotes per 100 cells were estimated. As exemplified in Fig. 7, CK18 RNAi-treated HeLa cells had lower numbers of amastigotes of Y or CL-Brener strains (Fig. 7C and Fig. 7F).

Table 1 depicts the frequency of infected cells and the number of amastigotes per 100 cells. The percentages of infected cells were similar when we compared CK18 RNAi-treated or control HeLa cells. In contrast, the number of amastigotes of Y or CL-Brener strains per 100 cells was significantly lower in HeLa cells transfected with CK18 RNAi when compared to controls.

**Table 1:** RNAi – medited knockdown of CK18 inhibits intracellular multiplication of amastigotes of Y or CL-Brener strains

**Parasite Strain**	**% of infected cell**			**Amastigotes/100 cells**		
	**Medium**	**CK18 RNAi **^a^	**CTR RNAi **^a^	**Medium**	**CK18 RNAi **^a^	**CTR RNAi **^a^
**Y**	32.0 ± 4.95	30.0 ± 3.53	28.0 ± 2.83	578 ± 11.36	**142 ± 20.66**^b^	497 ± 16.04
**CL-Brener**	33.0 ± 2.0	35.0 ± 4.16	33.0 ± 4.04	411 ± 14.57	**176 ± 18.55**^b^	406 ± 16.92

^a ^HeLa cells were transiently transfected with CK18 or control RNAi 24 h earlier as described in Methods section. These cells were infected with culture trypomastigotes of Y or CL-Brener strain using the parasite per cell ratio of 10:1 or 5:1 respectively. The number of intracellular amastigotes was estimated 48 h post-infection and the results are expressed as average of triplicates ± SD. This experiment was performed 3 times with similar results.

^b ^The number of amastigotes in cultures containing medium or RNAi control are lower than the number of amastigotes in cultures containing Medium with control RNAi (P < 0.01 in all cases, One Way Anova).

**Figure 8:**
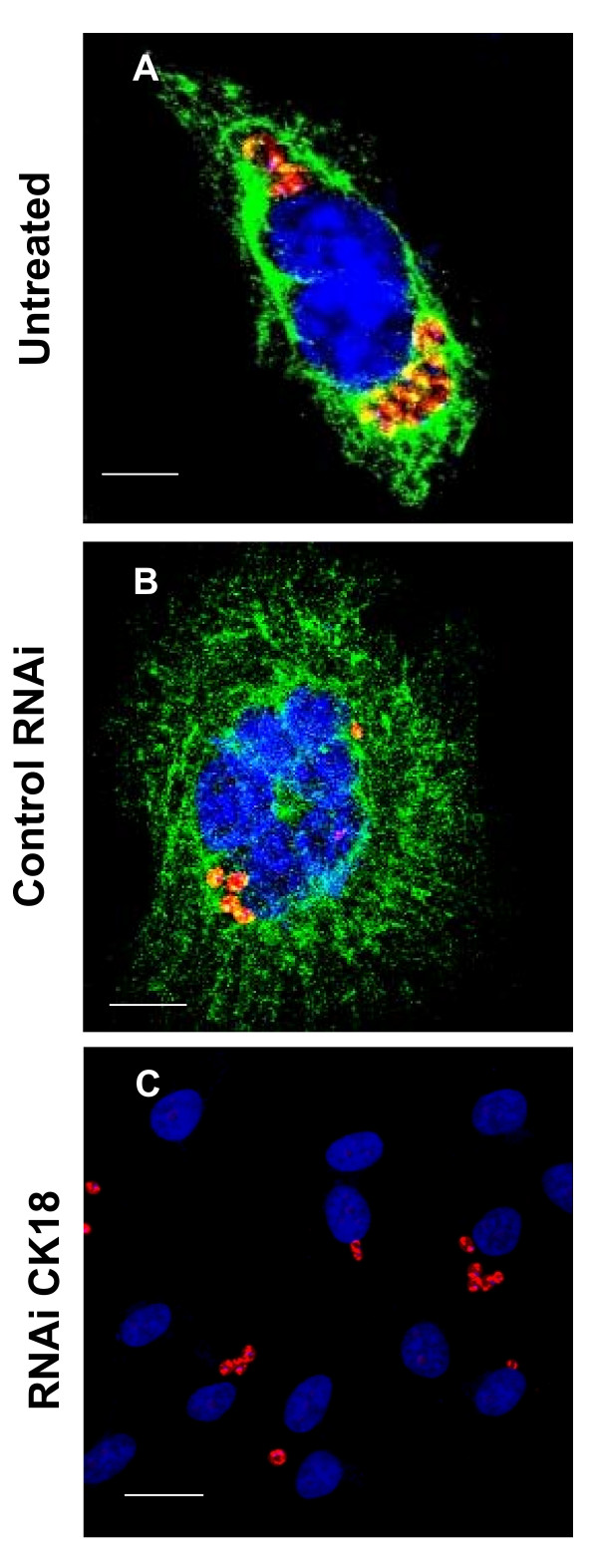
**Co-staining of intracellular amastigotes and CK18 in infected HeLa cells**. HeLa cells were left untreated (Medium, A) or transiently transfected with control RNAi (B) or CK18 RNAi (C). After 24 h, cells were infected with tissue culture trypomastigotes of the Y (A-C) strain. After 48 h, the cells were fixed, and stained for CK18 (green fluorescence), parasites (red fluorescence), and DAPI (blue). Images were obtained individually and merged. Magnification bar = 80 μM.

Because reduced levels of CK18 expression are, in some way, interfering in the intracellular amastigotes growth, we decide to evaluate whether there was a correlation between the cellular localization of CK18 and the parasite after infection. Co-localization experiments are depicted in Fig. 8. In HeLa cells infected with amatigotes of Y strain, we observed yellow staining denoting a merge between the green (CK18) and red (parasite) fluorescences (Fig. 8A and 8B). Treatment with CK18 RNAi strongly inhibited CK18 expression and reduced growth of amastigotes of the Y strain (Fig. 8C).

The experiment of co-localization described above suggested a physical interaction between amastigotes and HeLa cell's CK18. A putative parasite ligand for CK18 was described as the highly conserved FLY domain (VTVxNVxLYNR). ASP-2 expressed by amastigotes of the Y strain contains the FLY domain and could be the ligand to the host cytoplasmatic CK18 [17,24,25]. We performed a direct binding assay of the ASP-2 recombinant protein of Y strain (His-65 kDa, [17,25]) to fixed, permeabilized or not, HeLa cells *in vitro*. Although MAb to CK-18 strongly stained permeabilized HeLa cells (positive control), the recombinant protein His-65 kDa only binds to HeLa cells fixed but not permeabilized, suggesting a binding to the surface membrane (data not shown). In any case, we considered this binding irrelevant to our purposes because it was not inhibited by CK-18 RNAi treatment of HeLa cells (data not shown).

## Discussion

The present study was designed to evaluate the possible role of CK18 in the interaction between *T. cruzi *and non-phagocytic cells *in vitro*. The success of the transient knockdown of CK18 in cultured HeLa cells allowed us to further investigate for this topic. Unexpectedly, in the presence of limited amounts of CK18, trypomastigotes invaded HeLa cells efficiently. In addition, the recruitment of lysosomes to the parasitophorous vacuole and the subsequent escape from the parasitophorous vacuole/phagolysosomes were not impaired in CK18 RNAi-treated cells.

Once the amastigotes were in the cytoplasm of HeLa cells treated with CK18 RNAi, the growth of parasites of two different strains (Y or CL-Brener) was severely arrested. Co-localization studies support the hypothesis of close contact between amastigotes of the Y strain and CK18 in the HeLa cell cytoplasm. Because HeLa cells have CK8, it would be possible that it replace CK18. However, we observed that the level of expression of CK8 was also reduced following CK18 RNAi treatment of HeLa cells (Fig. 2).

It would be possible that RNAi to CK-18 inhibited the expression of parasites CK. However, gene silencing with RNAi in *T. cruzi *can not be performed due to the fact that this system is not present in these cells. Two main points have been discussed to explain this absence: (1) the AGO1 homologue (protein belonging to RISC and showed to be present on *Trypanosoma brucei*, [27]) or any other gene involved in RNAi pathway are absent; (2) at some point during the evolution, the parasite acquired an RNAi repressor as a reflection of a successful viral attack (reviewed [28]).

Cytokeratins are the most abundant structural proteins of epithelial cells, organized as heterodimers and forming a network of 10–12 nm wide intermediate filaments (IF). HeLa cells express CK8 and CK18. The IF maintains cell integrity, protects from environmental stress and regulates cytoplasmic protein availability, leading to the regulation of cell signaling. Indeed, CK18 alterations may be one of several anti-apoptotic events [29]. However, in our experiment, we observed no significant induction of apoptosis in HeLa cells in the absence of CK18. It has been shown that CK18 but not CK8 is a substrate for caspase digestion during the course of epithelial cell apoptosis, and the activation of caspase 3 is strictly dependent on the presence of CK18 [30]. Based on that, we can speculate that the reduced levels of CK18 caused by CK18 RNAi, could affect the caspase 3 activation, that in the absence of its substrate would be in the inactive form and consequently the cell would not enter in the apoptosis process.

Another interesting question is how the interaction with CK18 or CK8 facilitates the multiplication of amastigotes. Because very little is known about the parasite requirements for amastigote proliferation in the host cell cytoplasm, this question has no simple answer. Because amastigotes are intracellular forms and need to proliferate, it is possible that they bind to CK18. The binding to CK18 would initiate a signaling cascade within the parasite, recruiting the signaling proteins required for its multiplication. Our hypothesis is based on previous observations. Goto *et al*. [30] described the induction of growth of *Leishmania (Leishmania) mexicana *amastigotes and promastigotes by insulin-like growth factor I (IGF-I). IGF-I from the host binds to a single-site receptor on both promastigote and amastigote forms of Leishmania, initiating a signaling cascade [31]. IGF-I induced tyrosine phosphorylation of parasite proteins with different molecular weights in both forms of the parasite; it increased the DNA synthesis rate and consequently, the number of parasites inside the host cells rose [30]. The putative signaling pathway originated from the interaction between CK18 from the host cell and a putative parasite receptor is currently under investigation.

Our work is the third example of the use of RNAi technology to characterize proteins that might be involved in *T. cruzi *interaction with host cells. This approach proved to be feasible in small target projects and it has been used in pilot studies to conduct a genome-wide RNAi screen. We have already initiated this type of study in partnership with a private research institute. In the case of other non-neglected pathogens such as *Mycobacteria *and HIV, this approach is already in use [31-35].

Although the RNAi knockdown of host cells is being performed only to a limited extent and *in vitro*, it may be feasible to use RNAi technology to inhibit pathogen multiplication in localized areas of a patient's body. In the case of *T. cruzi*, this strategy might be useful, along with other forms of local treatment such as the recently developed cell therapy [36,37].

## Conclusion

The data presented here demonstrate that CK18-RNAi treatment of HeLa cells does not affect *T. cruzi *trypomastigotes invasion, the recruitment of lysosomes to the parasitophorus vacuole and the subsequent escape to the cell cytoplasm. On the other hand, CK18 expression in HeLa cells is important for the intracellular amastigotes multiplication of Y and CL-Brener strains. The elucidation of mechanisms used by the parasites for its replication inside the cell cytoplasm may lead to new paths for drug intervention.

## Methods

### Parasites and cells

Trypomastigotes of *T. cruzi *of Y strain were obtained from Dr. S. Schenkman (Universidade Federal de São Paulo). CL-Brener parasites were obtained from Dr. N. Yoshida (Universidade Federal de São Paulo). Parasites were maintained in cultured LLC-MK_2 _cells (also provided by Dr. S. Schenkman). All strains were molecularly typed based on small subunit rDNA sequences to confirm their origin [35]. These strains belong to diverse lineages. Y and CL-Brener strains are *T. cruzi *II and a hybrid I/II, respectively. HeLa cells provided by Dr. N. Yoshida were used for the *in vitro *experiments.

### Generation of double stranded RNAi and cell-transfections

The oligonucleotide retriever program available at  was used to generate sequences that code for RNAi of CK18 gene [GenBank: BC000180]. The oligonucleotides were synthesized (Stealth RNAi – Invitrogen). As negative control, we used Universal RNAi Stealth (Invitrogen, Nr lot 316294). HeLa cells were used for transfection experiments. Twenty four h before transfection, 1.5 × 10^4 ^cells were adhered to 24-well Costar plates in a final volume of 0.5 mL at 30–50% confluency. For transfection, 1 μL of Lipofectamin 2000 (Invitrogen) was admixed with RNAi (20 pmol) in a total volume of 100 μL of plain RPMI medium (Invitrogen) without serum or antibiotics. After 20 min rt, the mixture was added to the cells and incubated for 6 h. Transfection medium was removed and complete RPMI medium (cRPMI, RPMI supplemented with 10 mM HEPES, 24 mM sodium bicarbonate, 10% (vol/vol) of Fetal Bovine Serum (FBS, all purchased from Invitrogen), 100 U/ml of penicillin and streptomycin (Sigma)) were added.

Double strand RNAi sequences specific for CK18 gene used were:

i) RNAi: 5'-GCCCGUCUUGCUGCUGAUGACUUUA-3' (sense)

ii) RNAi: 5'-UAAAGUCAUCAGCAGCAAGACGGGC-3' (anti-sense)

### Real-time PCR quantification of CK18 transcripts

HeLa cells (10^7^) were washed with cRPMI medium and resuspended in the final volume of 0.25 mL. To this solution, we added 0.75 mL of Trizol (Invitrogen). Purified total RNA were treated with RNAse-free DNAse. First-strand cDNA was synthesized from 2 μg of purified total RNA using the ThermoScript RT-PCR System (Invitrogen) following the specifications provided by the manufacturer. Primers for reverse transcriptase reaction were oligo-DT provided with the kit. cDNAs were used for CK18 transcript quantification performed with the aid of the Applied Biosystems 7300 Real-Time PCR System. Reactions were performed in triplicate using SYBR Green (Invitrogen) in a final volume of 25 μl following the specifications provided by the manufacturer. The cycling conditions were 95°C for 2 min, 45 cycles of 95°C for 15 s, 60°C for 30 s and 72°C for 30 s. Melting curve analysis was performed heating the PCR products to 95°C and cooled down to 60°C. The quantity of RPL13 (housekeeping) transcript in each sample was used to normalize the amount of CK18 transcripts. The primers sequences used for quantitative Real-time PCR were:

i) RPL13A: 5'-CCTGGAGGAGAAGAGGAAAGAGA-3'(sense);

ii) RPL13A: 5'-TTGAGGACCTCTGTGTATTTGTCAA-3'(anti-sense);

iii) CK18: 5'-AATGGGAGGCATCCAGAACGAGAA-3'(sense);

iv) CK18: 5'-TTCTTCTCCAAGTGCTCCCGGATT-3'(anti-sense).

### Immunoblotting

HeLa cells (10^6^) grown in 6 wells plates (Costar), transfected or not with RNAi, were washed with cold PBS twice and lysed in the presence of lysis buffer prepared with sodium deoxicholate (0.25%, w/vol), HEPES (500 mM), NaCl (150 mM), Triton X-100 (1%, vol/vol), EGTA (20 mM), MgCl_2 _(1.5 mM), ortovanadate (100 mM), aprotinin (1 mg/mL), leupeptin (1 mg/mL), PMSF (100 mM), NaF (50 mM) and NaPyr (10 mM). Cell lysates were kept at -80°C for 24 h, centrifuged 15,000 g for 10 min and the supernatants were collected. Their protein content was estimated by the Bradford reagent (BioRad) and used to load SDS-PAGE. The volume equivalent to 10^6 ^lysed HeLa cells (~60 μg) were loaded onto 10% SDS-PAGE. Proteins were transferred to nitrocellulose membrane (Millipore) for 1 h at 100 V. Blocking solution was TBS containing 0.1% (vol/vol) of Tween and 2.5% (weight/vol) Bovine serum albumin (TBS-T/BSA) for 2 h at rt. After washed for 15 min with TBS-T, a monoclonal antibody to CK18 (Calbiochem) or β-actin (Sigma) were added at dilutions of 1:1,000 or 1:6,000, respectively, and incubated for 16 h at 4°C. After washes with TBS-T, peroxidase labeled goat anti-mouse IgG (Invitrogen) diluted 1:2,500 was added for 1 h at rt. After extensive washes, membranes were developed by enhanced-chemiluminescence system SuperSignal (PIERCE) and exposed to Hyperfilm (Kodak).

### Cellular infection assays

HeLa cells (1.5 × 10^4^) were plated in cRPMI medium in 24 well plates (Costar) containing 13-mm round coverslips at 37°C in a humidified atmosphere containing 5% CO_2_. These cells were transfected or not 24 h earlier with CK18-RNAi or control RNAi as described above. Parasite penetration to HeLa cells was evaluated at 2 h. Ten parasites per target cells was used for infection with trypomastigotes of the Y strain. In the case CL-Brener strain, we used 5 trypomastigotes per HeLa cell. To determine the number of parasites that invaded HeLa cells, we fixed them with a paraformaldehyde solution (2% vol/vol) for 30 min at rt. After washed twice with PBS and blocked with PBS-FBS (10% vol/vol) for 1 h a 37°C, we added rabbit polyclonal antibodies specific to *T. cruzi *(serum dilution of 1:200) followed by secondary staining with CY3-labeled goat anti-rabbit (1:50, Sigma) and DAPI (diluted 1:50, Invitrogen Molecular Probes). To estimate the number of parasites inside LAMP-1^+ ^vacuoles, cells infected at the indicated timing were fixed and subject to immunofluorescence assay (IFA) using a MAb specific for human LAMP-1 (1:500, HDB, Hybridome) and DAPI. Coverslips were washed in PBS, mounted and visualized under a fluorescence microscopy (Nikon E600). The number of internalized parasites was obtained by subtracting the number of bound CY3-fluorescent parasites from the total number of DAPI-stained kinetoplast DNA parasites per 100 HeLa cells. Parasite multiplication within cells was evaluated at 48 h using Giemsa stained coverslips and a conventional light microscope (Olympus CH30). The percentage inhibition of the amastigote growth was estimated by the formula: Number of amastigotes per 100 control cells – frequency of infected control cells/Number of amastigotes per 100 RNAi-treated cells – frequency of RNAi-treated infected cells. Infection assays using HeLa cells were done in triplicate and experiments were repeated in three or more occasion with similar results.

### Staining for CK8, CK18, CK19 and double-staining of parasites and CK18 in HeLa cells

HeLa cells (1.5 × 10^4^) were plated in cRPMI medium on 24 well plates containing 13-mm round coverslips at 37°C in a humidified atmosphere containing 5% CO_2_. These cells were infected with culture trypomastigotes of Y strain. The number of parasites per HeLa cell was 10:1, respectively. After 48 h, the cells were washed with PBS, fixed in paraformaldehyde solution (2% vol/vol) for 30 min at rt. After washed twice, the cells were permeabilized with PBS containing Triton X-100 (1% vol/vol) and blocked with PBS/BSA (3% weight/vol) for 40 min at rt. The cells were then stained with a MAb to CK8, CK18, CK19 (all purchased from Santa Cruz Biotechnology), or to CK18 (Calbiochem) diluted 1:40 in PBS/BSA for 1 h in a humid environment. Coverslips were washed with PBS and FITC-labeled anti-mouse IgG (1:100) added for 1 h. Coverslips were washed again and stained with the rabbit polyclonal antibodies specific for *T. cruzi *antigens (provided by Dr. S. Schenkman) diluted 1:100 for 1 h. After washes with PBS, the coverslips were incubated with CY3-labeled anti-rabbit IgG (Sigma) diluted 1:50 in PBS/BSA containing DAPI for 1 h. Coverslips were washed, mounted with buffered glycerin and observed on Confocal Microscope.

### Staining with Annexin-V-FITC/PI

HeLa cells (1.5 × 10^5^) were transiently transfected or not 24 h earlier with CK18-RNAi or control RNAi as described above. After that period, cell culture medium was exchanged and the cells cultured for additional 10 h in cRPMI medium. The cells were collected, washed in cold PBS and resuspended to a final concentration of 1 × 10^5^/ml in ligation buffer. 1 × 10^4 ^cells were labeled with FITC-conjugated Annexin-V (1:20) and propidium iodine (PI, 1:20 both from BD Biosciences Pharmingen) for 15 min in the dark. Cells were fixed with paraformaldehyde solution (2% weight/vol) and analyzed by fluorescence-activated cell sorting using FACSCanto (BD) and the software FlowJo (FlowJo).

### Statistical analysis

Values were compared by One-Way Anova followed by Tukey HSD tests available at the site . Differences were considered significant when the *P *value was < 0.05.

## Abbreviations

CK18: cytokeratin 18; CK19: cytokeratin 19; CK8: cytokeratin 8; RNAi: RNA interference; ASP-2: amastigote surface protein-2; PI: propidium iodine; IFA: immunofluorescence assay; IF: intermediate filaments; LLC-MK2: monkey rhesus kidney cell.

## Authors' contributions

CC carried out the molecular biology, microscopy and image analysis, and drafted the manuscript. MC carried out the Western blots. SMM and EVS performed experiments required by the reviewers. HPM and MMR supervised the design and coordination of all experiments and helped the final draft of the manuscript. All authors read and approved the final manuscript.
